# Long-term haplodeficency of DSPP causes temporomandibular joint osteoarthritis in mice

**DOI:** 10.1186/s12903-024-04320-8

**Published:** 2024-05-14

**Authors:** Qilin Liu, Yitong Zhao, Haibo Shi, Danwei Xiang, Chunye Wu, Lina Song, Ning Ma, Hongchen Sun

**Affiliations:** 1 Department of Oral and Maxillofacial Surgery, School and Hospital of Stomatology, Jilin University, Changchun, China; 2 Jilin Provincial Key Laboratory of Tooth Development and Bone Remodeling, School and Hospital of Stomatology, Jilin University, Changchun, China; 3 Department of Rheumatology, The First Hospital, Jilin University, Changchun, China; 4 Department of Oral Pathology, School and Hospital of Stomatology, Jilin University, Changchun, China

**Keywords:** Temporomandibular joint, Osteoarthritis, Mandibular condylar cartilage, Subchondral bone, Dentin sialophosphoprotein, Knockout mice

## Abstract

**Background:**

Extracellular matrix (ECM) protein malfunction or defect may lead to temporomandibular joint osteoarthritis (TMJ OA). Dentin sialophophoprotein (DSPP) is a mandibular condylar cartilage ECM protein, and its deletion impacted cell proliferation and other extracellular matrix alterations of postnatal condylar cartilage. However, it remains unclear if long-term loss of function of DSPP leads to TMJ OA. The study aimed to test the hypothesis that long-term haploinsufficiency of DSPP causes TMJ OA.

**Materials and methods:**

To determine whether *Dspp*^+/–^ mice exhibit TMJ OA but no severe tooth defects, mandibles of wild-type (WT), *Dspp*^+/–^, and *Dspp* homozygous (*Dspp*^*−/−*^) mice were analyzed by Micro-computed tomography (micro-CT). To characterize the progression and possible mechanisms of osteoarthritic degeneration over time in *Dspp*^+/–^ mice over time, condyles of *Dspp*^+/–^ and WT mice were analyzed radiologically, histologically, and immunohistochemically.

**Results:**

Micro-CT and histomorphometric analyses revealed that *Dspp*^+/–^ and *Dspp*^*−/−*^ mice had significantly lower subchondral bone mass, bone volume fraction, bone mineral density, and trabecular thickness compared to WT mice at 12 months. Interestingly, in contrast to *Dspp*^*−/−*^ mice which exhibited tooth loss, *Dspp*^+/–^ mice had minor tooth defects. RNA sequencing data showed that haplodeficency of DSPP affects the biological process of ossification and osteoclast differentiation. Additionally, histological analysis showed that *Dspp*^+/–^ mice had condylar cartilage fissures, reduced cartilage thickness, decreased articular cell numbers and severe subchondral bone cavities, and with signs that were exaggerated with age. Radiographic data showed an increase in subchondral osteoporosis up to 18 months and osteophyte formation at 21 months. Moreover, *Dspp*^+/–^ mice showed increased distribution of osteoclasts in the subchondral bone and increased expression of MMP2, IL-6, FN-1, and TLR4 in the mandibular condylar cartilage.

**Conclusions:**

*Dspp*^+/–^ mice exhibit TMJ OA in a time-dependent manner, with lesions in the mandibular condyle attributed to hypomineralization of subchondral bone and breakdown of the mandibular condylar cartilage, accompanied by upregulation of inflammatory markers.

**Supplementary Information:**

The online version contains supplementary material available at 10.1186/s12903-024-04320-8.

## Introduction

Temporomandibular joint osteoarthritis (TMJ OA) is a degenerative joint disease characterized by cartilage and subchondral bone damage as a result of irreversible degradation, destruction, or loss of extracellular matrix (ECM) components [1, 2]. Its causing factors include age, systemic illness, hormonal imbalances, aberrant mechanical loadings such as malocclusion, and genetic disorders, all of which appear to have a bearing on it [3, 4].

During the TMJ OA, the condyles in the TMJ complex are most preferentially affected and are therefore most extensively studied. Histologically, the mandibular condyle consists of subchondral bone and articular cartilage. The latter is fibrocartilage, containing ECM proteins and proteoglycans that provide the necessary properties to support the loads on the condyle [5]. Genetic alterations in the cartilage ECM proteins and secondary ECM breakdown play an essential role in TMJ OA initiation and disease progression [5]. On the other hand, the subchondral bone provides mechanical support for overlying articular cartilage and is responsible for the homeostasis and integrity of articular cartilage [6]. Abnormal catabolic signaling and/or low quality of subchondral bone could initiate the TMJ OA pathogenesis [6, 7]. For instance, biglycan and fibromodulin double-deficiency (*Bgn*^*−/0*^* Fmod*^*−/−*^*)* in mice not only disrupted chondrogenesis and ECM integration of mandibular condylar cartilage (MCC) [8] but also ruined subchondral bone integrity and turnover [1] in different stages of TMJ OA.

Besides proteoglycans such as biglycan and fibromodulin [9, 10], MCC also contains type I collagen, which is unique for fibrocartilage, along with other collagens including type II collagen, type IX collagen and type X collagen. Many genetic animal models with mutations in, or deletion of the above ECM components exhibit TMJ OA [5, 9–12].

DSPP belongs to the Small Integrin-Binding LIgand, N-linked Glycoprotein (SIBLING) family [13], which was identified in the MCC ECMs a decade ago. Other SIBLING family members include dentin matrix protein 1 (DMP1), bone sialoprotein (BSP), osteopontin (OPN), and extracellular matrix phosphoprotein (MEPE) [11]. Besides cartilage, DSPP is expressed in many other tissues such as dentin [14] and bone [15]. Indeed, DSPP is the most abundant non-collagenous protein in dentin. Genetic studies have demonstrated that *Dspp*^*−/−*^ (*Dspp* KO) mice manifested severe tooth defects resembling human dentinogenesis imperfecta III [14]. However, the amount of DSPP in cartilage and bone is relatively lower. For instance, in the long bone, the level of DSPP is only about 1/400 of that in dentin [15]. In spite of this, *Dspp*^*−/−*^ mice also exhibited hypomineralization in the long bone. Except for long bone, DSPP deletion also undermines calvaria and alveolar bone health [16, 17].

Due to the fact that DSPP deletion lessens MCC thickness by slowing down cartilage cell proliferation and jeopardizes the expression and distribution of other extracellular matrices, such as collagen II, IX, X, as well as biglycan [18]. We concluded that DSPP played an essential role in MCC development and maintenance, and hypothesized that long-term deficiency of DSPP might lead to TMJ OA-like MCC degeneration and subchondral bone damage. However, *Dspp* null mice bear severe tooth defects including pulp exposure, tooth loss, and periodontal destruction [14] which may severely disturb the occlusal balance and lead to secondary structural changes of the mandibular condyle. As *Dspp*^+/–^ mice have no significant tooth phenotype [19–21], we employed *Dspp*^+/–^ (heterozygous *Dspp* KO) mice to examine the long-term effects of DSPP haplodeficency in MCC and subchondral bone, to check the direct function of DSPP on condyle excluding occlusal effect. We found that *Dspp*^+/–^ mice exhibited marked subchondral bone destruction similar to *Dspp*^*−/−*^ mice when compared to WT littermates. In contrast to WT mice, we looked for signs of TMJ OA in the overtly normal heterozygous *Dspp* KO mice. The evidence of fibrillation of the articular surfaces, fissuring of the cartilage, and ultimately separation of articular cartilage and subchondral bone degeneration were found through 12 months to 18 months and presented as a gradual process over age. Thus, this study was able to demonstrate that DSPP haplodeficency accelerated the osteoarthritis-like lesions in mandibular condyle.

## Materials and methods

### Animal preparation

Homozygous *Dspp* global knockout mice, generated in NIDCR [14], in the C57BL/6 background (referred to as *Dspp*^*−/−*^ mice hereafter) were employed. The heterozygous *Dspp* knockout mice (*Dspp*^+*/–*^ mice) were obtained by *Dspp*^*–/–*^ mice crossbred with C57BL/6 wild-type mice. The 4-month, 8-month, 12-month, 15-month, 18-month, and 21-month-old mice (*n* = 3 in each group) were obtained and divided into three groups: the WT group, the *Dspp*^+/–^ group, and the *Dspp*^+/–^ group. Food and water were given as usual. All mice were sacrificed, and the mandibles were dissected at a well-designed time under sevoflurane anesthesia.

### Sample preparation

Mandibles from WT, *Dspp*^*−/−*^ and *Dspp*^+/–^ mice were dissected. Half of the mandible from each mouse was fixed in 4% paraformaldehyde overnight, then transferred into 70% ethanol and stored at 4 °C for plain X-ray and 3D micro-computed tomography (micro-CT) analysis. The remaining half mandible was fixed in 4% paraformaldehyde for 24 h, then decalcified in a 15% EDTA solution for 6 weeks at 4 °C, followed by embedding in paraffin after the mandibular body was cut off at the condylar neck. 3-μm-thick of sagittal sections were used for hematoxylin and eosin (HE) staining, toluidine blue staining, Safranin O staining, Masson staining, tartrate-resistant acid phosphatase (TRAP) staining, and immunohistochemical staining.

### X-ray radiography imaging analysis

Every three mandibles from WT mice and *Dspp*^+/–^ KO mice at the age of 12 months were selected and analyzed with the Faxitron MX-20DC12 Specimen Radiography System (Faxitron X-ray Corp., Buffalo Grove, IL, USA).

### Three-dimensional micro-computed tomography (μCT) analysis

Three-dimensional images of the mandibles were taken using μCT50 (Scanco Medical AG, Bassersdorf, Switzerland). The following parameters of voxel size 10 μm, 200 mA, 70 kVp, and exposure time 300 ms were used during scanning. After reconstruction, data were analyzed with the 3D-Creator software supplied with the instrument, and the subchondral bone structural analysis was conducted. Morphometric parameters including bone volume (BV), total volume (TV), bone volume fraction (BV/TV), porosity (1-BV/TV), bone mineral density (BMD), tissue mineral density (TMD), trabecular number (Tb. N), trabecular thickness (Tb. Th), trabecular separation (Tb. Sp) and degree of anisotropy (DA) were calculated. Using InVivo Dental 5.0 software (Anatomage, San Jose, CA, USA), three-dimensional multiplanar reconstruction (MPR) of mandibular condyles was surveyed in detail.

### Histochemical and immunohistochemical staining

HE and toluidine blue staining were performed on mouse condylar sections at all time points of 12, 15, and 18 months. For 12-month-old mice, the MCC cell numbers were counted based on HE and toluidine blue-stained cross sections and used for statistical analysis. According to the modified Mankin scoring system [22], we graded the pathologic condition of the MCC in four categories: cartilage erosion score, chondrocyte periphery staining, spatial arrangement of chondrocytes, and background staining intensity. For the 15- and 18-month condylar sections, HE, toluidine blue, Safranin O, and Masson stain were applied. To assess osteoclast differentiation, paraffin slides were stained for tartrate-resistant acid phosphatase (TRAP) (Sigma, St. Louis, MO, USA). TRAP-positive cells with three or more nuclei were counted as osteoclasts. Condylar sections of 8-months mice were selected for immunohistochemical staining, antigen retrieval was performed in pepsin antigen retrieval solution (MXB, Fuzhou, China) using water-bath heating at 50℃. The antibodies used in the immunohistochemical analyses were FN1 (Sangon Biotech, Shanghai, China), TLR4 (Sangon Biotech, Shanghai, China), MMP2 (Sangon Biotech, Shanghai, China) and IL-6 (Sangon Biotech, Shanghai, China). All antibodies had a dilution ratio of 1:200 with 2% BSA. The immunohistochemical kit (MXB, Fuzhou, China) was used according to the instructions. Finally, the sections were counterstained with hematoxylin.

### RNA sequencing

TMJ condyles with an intact cartilage/subchondral bone interface were dissected from 4-month-old WT and *Dspp*^+/–^ KO mice. Tissues from 3 independent animals from WT or *Dspp*^+/–^ KO mice were pooled to create a single test sample. RNA extraction, library construction, sequencing, and data analysis were performed by CapitalBio Technology (Beijing, China).

### Statistical analysis

All data were presented as mean ± standard deviation (SD) and analyzed with Kruskal–Wallis Test using SPSS 16.0 software (SPSS). Differences with a *p-value* < 0.05 were considered statistically significant.

## Results

### DSPP haplodeficency, like the total loss-of-DSPP, led to subchondral bone hypomineralization of the mandibular condyle

**Fig. 1:**
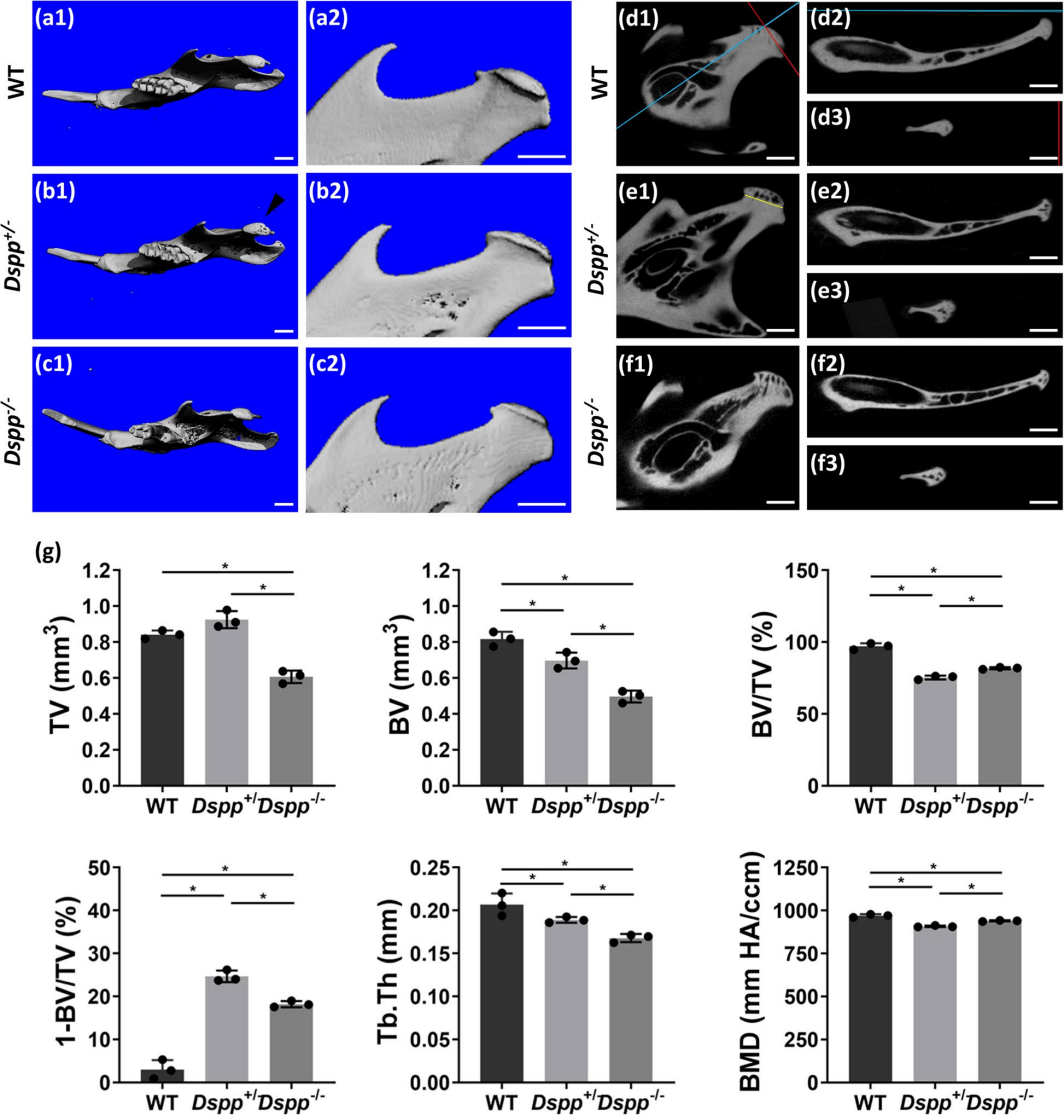
Mandible condylar structure changes in *Dspp*^+/–^ and *Dspp*^*−/−*^ mice compared to WT mice. (a1, b1, c1) Top view of micro-CT reconstructions of mandibles respectively from 12-month-old WT, *Dspp*^+/–^, and Dspp^−*/−*^ mice. Note that the condylar surface of *Dspp*^+/–^ mice was coarse (denoted as black arrowhead). (a2, b2, c2) Side view of micro-CT reconstructions of condyle from 12-month-old WT, *Dspp*^+/–^ and *Dspp*^*−/−*^ mice. (d1, e1, f1) Sagittal planes of 3-dimensional multi-planar reconstruction (3D-MPR) images of mandibular condyles from 12-month-old WT mice, *Dspp*^+/–^ and *Dspp*^*−/−*^ mice. A blue line and a red line in d1 represented the cut planes in d2 and d3, respectively. (d2, e2, f2) Coronal cutting planes of 3D-MPR images. (d3, e3, f3) Axial cut planes of 3D-MPR images. (g) Quantification of the parameters concerning the subchondral bone by micro-computed tomography (micro-CT). Note that the chosen area was defined as the subchondral bone area located above the yellow line, as illustrated in e1. Values were presented as mean ± SD. *n* = 3 per group, *P < 0.05. TV, tissue volume; BV, bone volume; BV/TV, bone volume fraction; 1-BV/TV, bone porosity; BMD, bone mineral density; Tb.Th, trabecular thickness. SD, standard deviation

The mandibles of 12-month-old mice in all three groups were measured and reconstructed by micro-CT, with images presented either in top view or side view (Fig. 1a1-c1, a2-c2). Data of homozygous deletion of DSPP showed that loss of DSPP led to tooth loss in *Dspp*^*−/−*^ mice at 12 months (Fig. 1c1), while teeth of *Dspp*^+/–^ mice were intact, without significant differences from WT mice at the same age (Fig. 1a1, b1). Interestingly, the condylar surface of *Dspp*^+/–^ was coarse (Fig. 1b1), which indicated subchondral bone destruction.

To check in detail the subchondral bone mineralization of mandibular condyles of the three groups at the age of 12 months, 3D-MPR was employed (InVivo Dental 5.0, Anatomage, San Jose, CA, USA). Sagittal sections of the micro-CT images (Fig. 1d1, e1, f1) showed that there were big subchondral bone trabecular spaces in 12-month-old *Dspp*^+/–^ and *Dspp*^*−/−*^ mice compared to WT mice at the same age. Additionally, horizontal and coronal planes showed that the condylar subchondral bone, and the condylar necks in *Dspp*^+/–^ and *Dspp*^*−/−*^ mice were more porous than those in WT mice (Fig. 1d2, d3, e2, e3, f2, f3). Interestingly, there was no marked difference in mineralization between *Dspp*^+/–^ and *Dspp*^*−/−*^ mice, which indicated that DSPP haplodeficency, similar to DSPP total deletion, led to less bone mass and less mineralization in the subchondral bone of mandibular condyle.

To quantitatively measure the mineralization of the subchondral bone, three-dimensional morphometric parameters were analyzed. To standardize measurement, the subchondral bone area located above the yellow line was selected for assessment, as depicted in Fig. 1e1. The chosen area was characterized by an intact cartilage/subchondral bone interface, which ensured consistency and accuracy in the evaluation of the samples, and allowed for reliable comparisons between the different groups. Data showed that there were significant reductions in the BV, BV/TV, BMD, and Tb. Th in *Dspp*^+/–^ and *Dspp*^*−/−*^ mice compared to WT mice. Contrary to BV/TV, the subchondral bone porosity significantly increased in *Dspp*^+/–^ and *Dspp*^*−/−*^ mice (Fig. 1g). Unexpectedly, the subchondral bone porosity in *Dspp*^+/–^ mice was more severe, while bone mineral density in *Dspp*^+/–^ mice was less than in *Dspp*^*−/−*^ mice (Fig. 1g). On the contrary, the increased porosity in the condylar neck region of *Dspp*^*−/−*^ mice was more remarkable compared to *Dspp*^+/–^ based on 3D-MPR (Fig. 1e2 and f2). These results suggest that hypomineralization occurs with deletion in either one allele or both alleles of *Dspp*, even though it is non-equalized in the condylar neck and subchondral bone.

To examine how the haploinsufficiency of DSPP affects osteoblastic and osteoclastic differentiation, we perform RNA sequencing, data showed that haplodeficency of DSPP affects the biological processes of ossification and osteoclast differentiation (Fig.s1). In addition, the data presented in (Fig.s2) illustrates the relative amount of DSPP expression in the condyles of both WT mice and *Dspp*^+/–^ mice. It is worth noting that the *DSPP* in *Dspp*^+/–^ mice was comparatively weaker than that in WT mice. This information can be used constructively to further understand the role of DSPP in the development of the condyles and its contribution to healthy joint function.

Taken together, both *Dspp*^+/–^ and *Dspp*^*−/−*^ mice exhibited subchondral bone hypomineralization, including increased porosity and reduced BMD. Given the non-negligible effect of occlusion disorder on TMJ in *Dspp*^*−/−*^ mice, the heterozygous *Dspp* KO mice were employed as the animal models to examine the direct role of DSPP on mandibular condyles.

### DSPP haplodeficency led to TMJ-OA-like lesions with MCC attenuation and subchondral bone hypomineralization

**Fig. 2:**
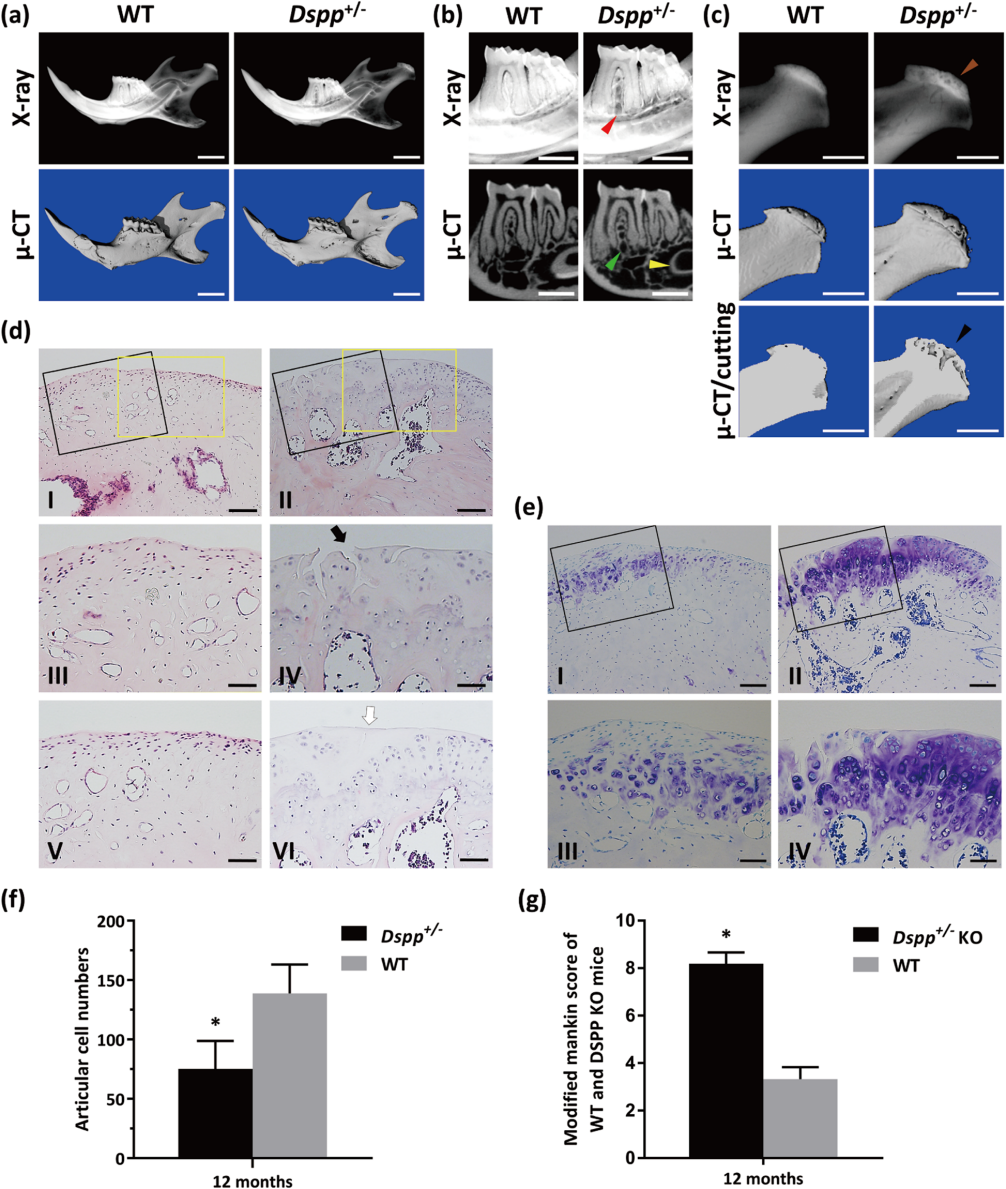
TMJ-OA-like changes in MCC and subchondral bone in *Dspp*^+/–^ mice. **a** X-ray and micro-CT images of mandibles from 12-month-old WT and *Dspp*^+/–^ mice. **b** Close-up views of the molars in a. Note that there were no significant changes in the tooth bodies between the two groups, except for slight crown attrition, decrease of bone density around tooth root in *Dspp*^+/–^ mice (denoted as red and green arrowheads) and thinner dentin in incisor root (denoted as a yellow arrowhead). **c** Respective close-up views and cross-sections of condyles in a. Note that translucent areas could be observed in the subchondral bone of *Dspp*^+/–^ mice compared to WT mice (denoted as a brown arrowhead); additionally, the condylar surface was irregular in *Dspp*^+/–^ mice. **d** H&E staining of mandibular condyles from 12-month-old WT and *Dspp*^+/–^ mice. III and VI are higher magnification views of the black boxed area in I and II; V and VI are higher magnification views of the yellow boxed area in I and II. The articular fissures (denoted as black arrow) and acellular areas (denoted as white arrow) of the MCC were remarkable in *Dspp*^+/–^ mice. **e** Toludine staining of mandibular condyles from 12-month-old WT and *Dspp*^+/–^ mice. III and VI are higher magnification views of the black boxed area in I and II. The articular fissures (denoted as black arrow) and acellular areas (denoted as white arrow) of the MCC were remarkable in *Dspp*^+/–^ mice. Scale bars: 100 μm for lower magnification, 50 μm for higher magnification of the boxed areas. **f** Statistics of articular cell numbers. The cells in the MCC decreased markedly in *Dspp*^+/–^ mice compared to WT mice. **g** Statistics of the modified Mankin score. The score was significantly elevated in the *Dspp*^+/–^ mice. Values were presented as mean ± SD. *n* = 3 per group, *P < 0.05. SD, standard deviation

To further assess the mandible phenotype underlying *Dspp* heterozygous deletion in detail, we first employed plain x-ray and micro-CT to analyze the overall tooth and condyle structures of mandibles of *Dspp*^+/–^ mice and WT mice at 12 months (Fig. 2a, b, c). Images showed that there was no apparent tooth body defect in *Dspp*^+/–^ mice in comparison with WT mice (Fig. 2a, b). However, decreased BMD of the mandibular condyle can be observed in *Dspp*^+/–^ mice compared to WT mice on enlarged x-ray images (Fig. 2c). In micro-CT images, the condylar surface was more coarse in 12-month-old *Dspp*^*−/−*^ mice than in WT mice (Fig. 2c). Moreover, increased porosities, indicating hypomineralization, of condylar subchondral bone in *Dspp*^+/–^ mice were observed (Fig. 2c).

To determine whether one allele deletion of *Dspp* will affect MCC, we performed the hematoxylin and eosin (H&E) and toluidine blue (TB) staining of representative MCC specimens from WT and *Dspp*^+/–^ mice at the age of 12 months (Fig. 2d, e). Data showed that *Dspp*^+/–^ mice exhibited apparent fissures and decreased chondrocyte cells in MCC accompanied by some large cavities in the subchondral bone. Data statistics proved that MCC of *Dspp*^+/–^ mice had a reduced number of MCC cells compared to WT mice (Fig. 2f). The decreased cell amounts found in *Dspp*^*−/−*^ mice might also be attributed to the impeding proliferation of MMC cells as we demonstrated in *Dspp*^*−/−*^ KO mice [17]. The modified Mankin scores showed that the haplodeficency of DSPP could lead to osteoarthritis (Fig. 2g).

### TMJ OA severity caused by DSPP haplodeficency was positively correlated with age

**Fig. 3:**
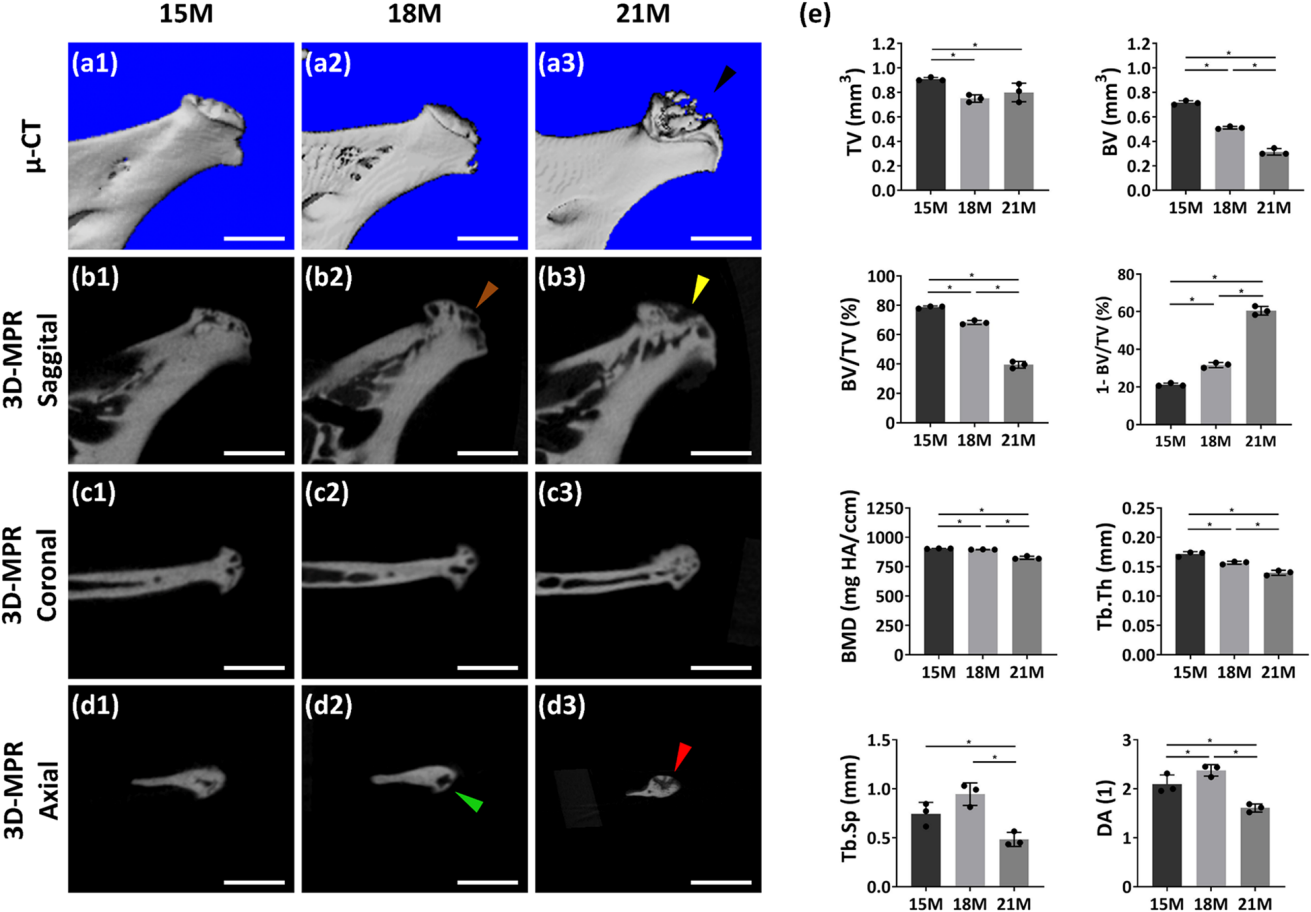
TMJ OA changes with aging in *Dspp*^+/–^ KO mice. (a1-a3). Morphologic observation of the mandibular condylar cartilage from *Dspp*^+/–^ mice based on the reconstruction of micro-computed tomography (micro-CT) at the age of 15 months, 18 months, and 21 months, respectively. Black arrowhead is pointing the condylar malformation. (b1-b3). Sagittal view of 3-dimensional multi-planar reconstruction (3D-MPR) images of mandibular condyles in *Dspp*^+/–^ KO mice. At the age of 18 months, bone cavities were more visible than at 15 months. Plus, the cavities were closer to the bone surface (denoted as a brown arrowhead). Note that in condyles of 21 months, open cavities could be observed, which was a mark of bone collapse and osteophytes (denoted as a yellow arrowhead). (c1-c3) and (d1-d3) are the coronal view and axial view of 3D-MPR images from corresponding samples. We can see the bone cavities were very near to the bone surface at 18 months (green arrowhead). At 21 months, osteophytes could be visualized (red arrowhead). Scale bars = 1 mm in all images. (e). Subchondral bone parameters based on micro-CT were statistically analyzed. Values were presented as mean ± SD. *n* = 3 per group, *P < 0.05. TV, tissue volume; BV, bone volume; BV/TV, bone volume fraction; 1- BV/TV, porosity; BMD, bone mineral density; Tb.Th, trabecular thickness; Tb.Sp, trabecular separation; DA, degree of anisotropy; SD, standard deviation

To determine whether TMJ OA caused by DSPP haplodeficency is age-related, we first performed micro-CT scans on mandibles of 15-, 18-, and 21-month-old heterozygous *Dspp* KO mice. The condyles from *Dspp*^+/–^ mice showed irregular condylar surfaces on the side-view reconstructed images. Notably, the osteophyte formation could be seen in 21-month-old mice (Fig. 3a3). 3D- MPR of micro-CT data exhibited that trabecular bone spaces accelerated as mice age. Besides, based on micro-CT quantification analysis, we found that BV, BV/TV, BMD, and Tb.Th decreased with age, but the subchondral bone porosity increased with age (Fig. 3e). Tb.Sp reduced at 21 months in comparison with 15 months and 18 months (Fig. 3e). This may be correlated to osteophyte formation, which also influences mouse chewing. The degree of Anisotropy (DA) indicates trabecular structure diversities associated with loading and stress [23]. Elevated DA suggests an increase in osteoporosis. Our data showed DA kept increasing from 15 to 18 months, but decreased at 21 months (Fig. 3e). This may be an adaptation of mechanical alteration due to condylar malformation and successive mastication abnormality. Therefore, our findings showed statistically significant changes in the subchondral bone of the condyles at these three time points (Fig. 3).

**Fig. 4:**
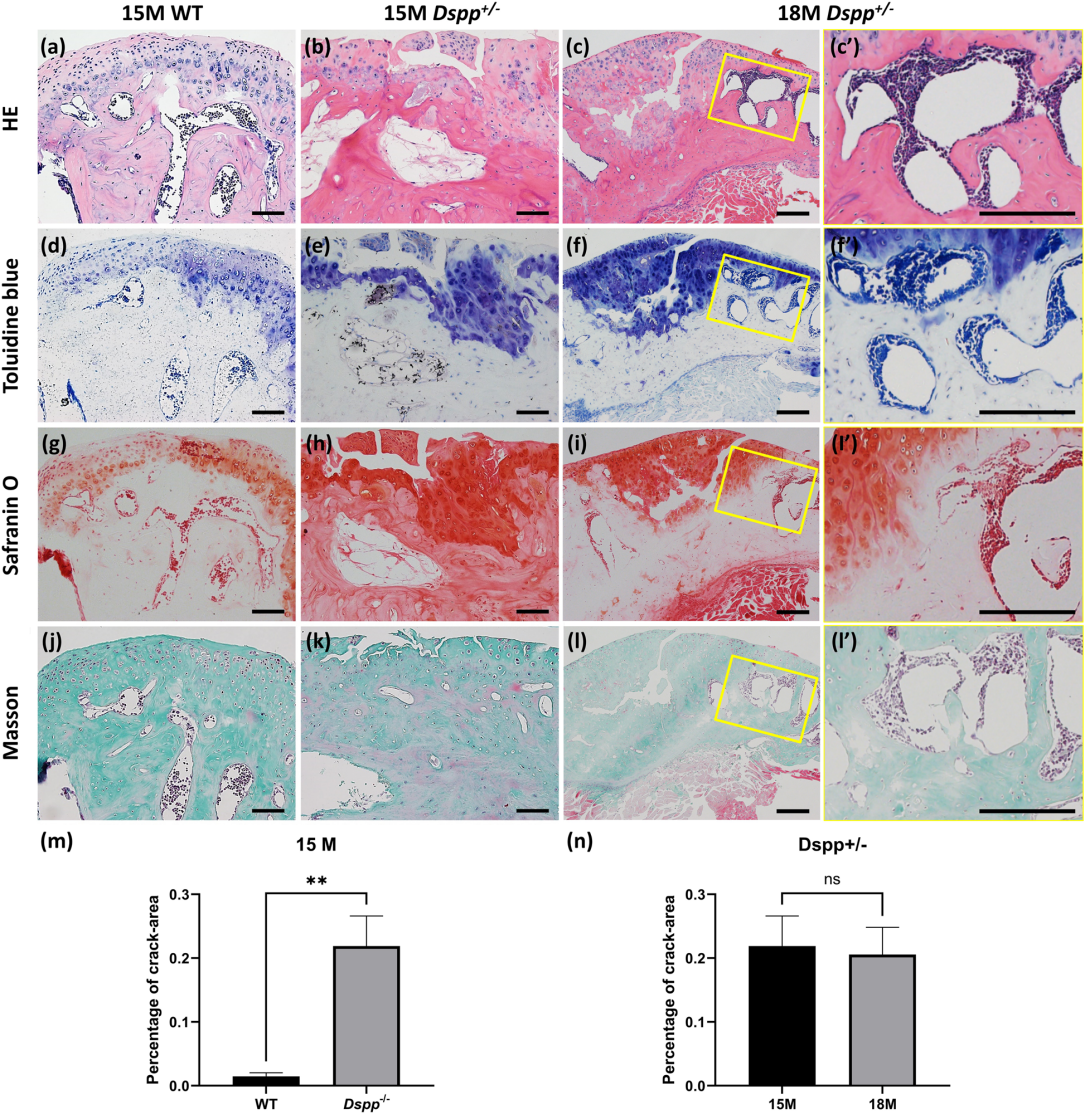
Assessment of cartilage breakdown and bone resorption in the MCC from the 15-month and 18-month-old *Dspp*^+/–^ mice. H&E (**a**-**c**), toluidine blue (**d**-**f**), safranin O staining (**g**-**i**) and Masson staining (**j**-**l**) of condyle samples collectively showed that cartilage fissures in *Dspp*^+/–^ mice, while no apparent cracks in that of. (c’, f’, i’, l’) are high magnification views of the yellow boxed area in (c, f, i, l), respectively. Bars = 200 μm. Statistical results (**m**, **n**) display the percentage of crack-area in condyles of 15-month-old WT mice and 15/18-month-old *Dspp*^+/–^ mice. Values are presented as mean ± SD. *n* = 3 per group, **P < 0.01

To assess the pathological changes of cartilage and subchondral bone caused by long-term haplodeficency of DSPP, a set of histochemical staining was performed. The HE staining data showed severe cartilage fissures, acellular zones of cartilage, and big subchondral cavities in *Dspp*^+/–^ mice, with some cartilage debris peeling off the cartilage (Fig. 4a-c). Toluidine blue, Safranin O, and Masson staining of the condyle sections confirmed cartilage degradation and subchondral bone defects in *Dspp*^+/–^ mice (Fig. 4d-l). In condyles from 18-month-old *Dspp*^+/–^ mice, the cartilage fissure deepened into the cartilage-bone interface and exhibited regional loss of chondrocytes, subchondral bone erosion, as well as uneven staining in the cartilage matrix (Fig. 4c–c’, f-f’, i-i’, l-l’). The statistical findings (Fig. 4m, n) suggest that the integrity of the condyle structure in *Dspp*^+/–^ mice has undergone significant deterioration when compared to that of the WT mice. Specifically, the results indicate that the condyles in *Dspp*^+/–^ mice have been severely damaged. Such findings may have important implications for our understanding of the underlying mechanisms contributing to the observed structural differences between the two groups. Further research is warranted to elucidate the potential clinical significance of these findings.

In general, the phenotypes of condylar cartilage and subchondral bone caused by heterozygous deletion of *Dspp* deteriorated with age. It was worth noting that all teeth of *Dspp*^+/–^ mice at any of the time points appeared normal (data not shown), which further approved that *Dspp*^+/–^ mice were a reliable model for detecting DSPP deficiency role on MCC excluding occlusal factors.

### DSPP haplodeficency aggravated TMJ OA by increasing osteoclast distribution and upregulation of inflammatory markers

**Fig. 5:**
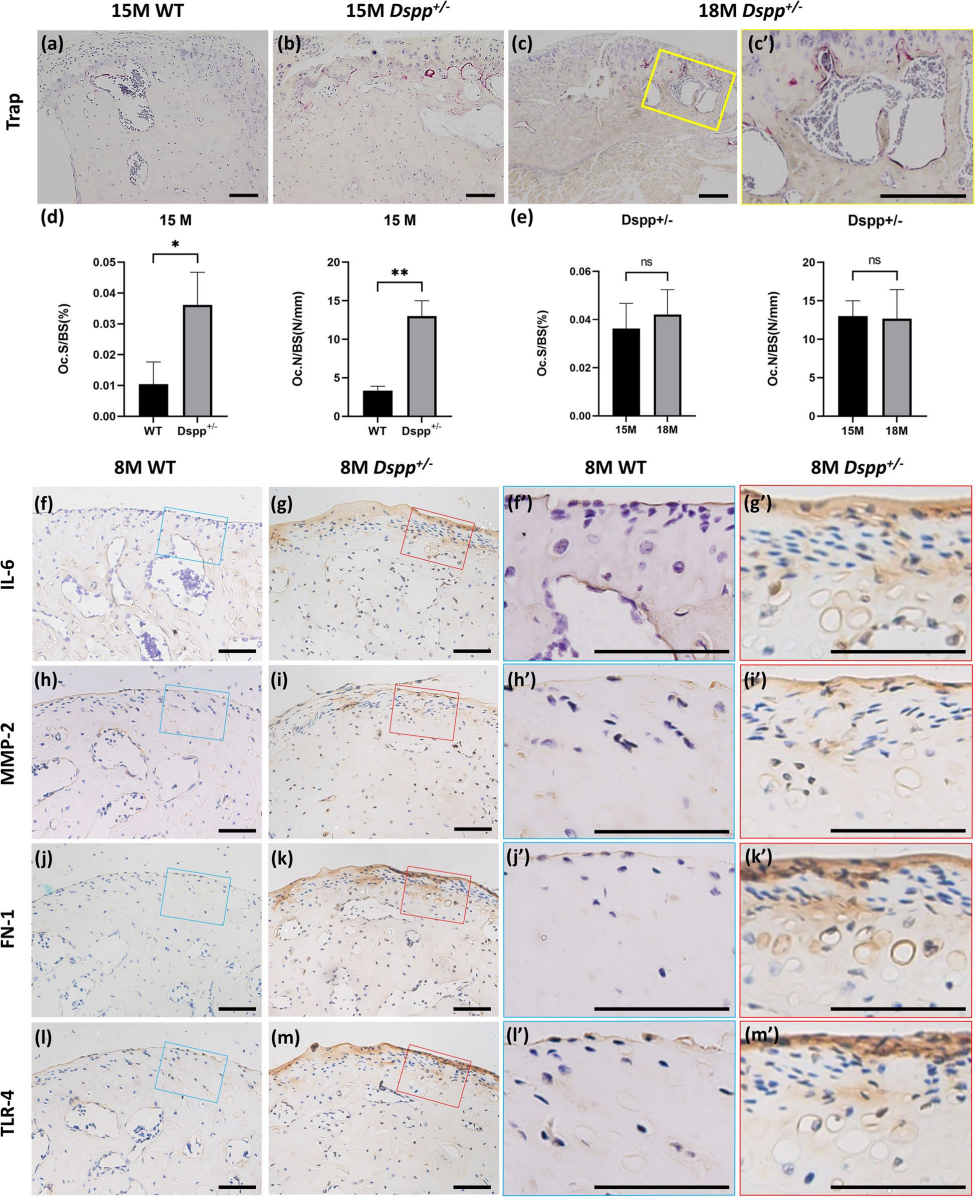
Tartrate-resistant acid phosphatase (TRAP) staining (**a**-**c**) showed that there were more positive cells on the edges of bone cavities in *Dspp*^+/–^ mice than in WT mice, which indicated upregulated osteoclast activities in the subchondral bone. (c’) is a high magnification view of the yellow boxed area in (**c**). Statistic quantitation to measure levels of TRAP-positive cells with no less than three nuclei (**d**, **e**). Oc. S/BS, osteoclast surface per bone surface. Oc. N/BS, osteoclast number per bone surface. Values are presented as mean ± SD. *n* = 3 per group, *P < 0.05, **P < 0.01. SD, standard deviation. **f**-**m** Alterations in the inflammatory markers and DAMPs-related factors in the mandibular condylar cartilage (MCC) from the 8-month-old *Dspp*^+/–^ mice. Immunoreactivity of IL-6 (**f**, **g**), MMP-2 (**h**, **i**), FN-1 (**j**, **k**) and TLR-4 (**l**, **m**) in the MCC was increased in *Dspp*^+/–^ mice compared to WT mice. (f’, h’, j’, l’) are higher magnification views of the blue boxed area in (**f**, **h**, **j**, **l**). (g’, i’, k’, m’) are higher magnification views of the red boxed area in (**g**, **i**, **k**, **m**). Scale bars, 200 μm

As the decreased bone volume and increased bone cavities in the subchondral bone of the condyle in *Dspp*^+/–^ mice was observed, we performed TRAP staining to assess osteoclast activities in the subchondral bone (Fig. 5a-c, c’). Fifteen-month-old data showed that osteoclast surface per bone surface (Oc. S/BS) and osteoclast number per bone surface (Oc. N/BS) was significantly increased in *Dspp*^+/–^ mice compared to WT mice. (*p* < 0.05, Fig. 5d). TRAP-positive cells in the subchondral bone were markedly elevated in *Dspp*^+/–^ mice, about three times over that of WT mice based on statistical quantitation analysis. In addition, we found that the Oc. S/BS and Oc. N/BS were insignificantly increased with age, as evidenced by the data from 15- and 18-month-old *Dspp*^+/–^ mice (Fig. 5e).

We also found that the inflammatory markers such as IL-6 and MMP-2 were increased in MCC of *Dspp*^+/–^ mice compared to WT mice at 8 months (Fig. 5f-g, f’-g’, h-i, h’-i’), which confirmed the inflammatory changes occurred in mandibular condyle. Moreover, reliable evidence suggests that Damage Associated Molecular Pattern (DAMP)-induced inflammation plays an important role in the pathogenesis of OA. FN-1, as a DAMP molecule, can induce activation of a toll-like receptor 4 (TLR4) and promote the release of inflammatory factors [24]. Therefore, to uncover the possible reason that DSPP haplodeficency led to TMJ OA, we observed the alteration of FN-1 and TLR-4 in MCC, data showed both molecules markedly increased in MCC (Fig. 5j-k, j’-k’, l-m, l’-m’).

## Discussion

MCC plays a pivotal role in buffering bone against stress [12]. Cartilage ECM, which comprises collagen and non-collagenous proteins, is the load-carrying structural component [25]. With aging, the homeostasis between the synthesis and degradation of several cartilage ECM components is disrupted. Hence, OA might be naturally developed with an extended lifespan [26–28] due to loss of cartilage integrity and progressive deterioration of its biomechanical properties [29]. Pathological changes in ECM amount, structure, or function were responsible for early-onset OA associated with variable degrees of cartilage degradation in human patients or animal models [8–10, 30–34].

Dr. Sun first discovered the presence of DSPP in cartilage [13, 35] and profiled DSPP expression in rat MCC [13]. We successively demonstrated that postnatal *Dspp*^*−/−*^ mice exhibited reduced MCC cell proliferation, cartilage thinning, and abnormal expression of other ECM including collagen type II, collagen type XI, collagen type X, and biglycan [17]. Based on our findings in *Dspp*^*−/−*^ mice up to 6 months, we speculate that long-term loss of DSPP may contribute to TMJ OA. However, due to the non-negligible impact of malocclusion, the pathogenesis of TMJ-OA may be clinically complicated or may be induced by multiple factors.

To understand the specific functions of DSPP on condyles, we employed *Dspp*^+/–^ mice to observe long-term DSPP haplodeficency on the condyle, because previous studies reported that *Dspp*^+/–^ mice have no teeth phenotype [36, 37]. Although recently we found that *Dspp*^+/–^ mice exhibited teeth phenotype like human DD-II, and had mild periodontitis [20], Our findings (Fig. 2b) showed that the tooth phenotype was too weak to impact occlusal balance compared with *Dspp*^*−/−*^ mice at the age of 12 months. Besides, no pulp exposure, tooth loosening, or loss occurred in *Dspp*^+/–^ mice at all the time points from 12 to 21 months in our study. Unexpectedly, the subchondral bone porosity in *Dspp*^+/–^ mice was more severe, while bone mineral density in *Dspp*^+/–^ mice was less than in *Dspp*^*−/−*^ mice. These results suggest that hypomineralization occurs with deletion in either one allele or both alleles of *Dspp*. On the other aspect, the severe tooth defect of *Dspp*^*−/−*^ mice may alter the mechanical environment of the condyle.

It has been reported that wild type C57BL/6J mice spontaneously develop temporomandibular arthritis at 60 weeks [28]. We therefore first selected wild-type mice at the age of 12 months and compared them to *Dspp*^+/–^ mice, which showed a more marked osteoarthritis phenotype than WT mice, suggesting that DSPP haplodeficiency accelerates and exacerbates the onset of osteoarthritis. In our *Dspp*^+/–^ mouse model, TMJ-OA was evidenced by articular cartilage degradation and abnormal subchondral bone remodeling and was also age-related. Pathological alterations including decreased thickness and hypocellularity of the cartilage layer, emerging articular cartilage fissures, and large subchondral bone cavities could be observed in HE, Toluidine blue, as well as Safranin O stained images. As the homozygous deletion of *Dspp* would decrease cartilage cell proliferation and lead to MCC thinning [17], we postulated that the downregulation of chondrocyte proliferation might partially cause cartilage thickness reduction in *Dspp*^+/–^ mice. Besides cellular hypocellularity, lack of DSPP and subsequent breakdown of other ECM components might lead to cartilage fissures [17]. In addition, cartilage fissures deep down to the cartilage-bone interface, where the subchondral bone was exposed to the articular cavity, might contribute to bone apophysis formation and condyle deformation, further exacerbated the symptoms of TMJ-OA, as visualized in representative mice at 21 months of age.

On the other hand, the cartilage breakdown may be exaggerated by abnormal subchondral bones. RNA sequencing data suggest that haplodeficency of DSPP affects the biological process of ossification and osteoclast differentiation. Micro-CT data of 12- to 21-months of *Dspp*^+/–^ mice collectively exhibited that large subchondral cavities. It is worth noting that the cavities move closer to the condylar surface with age. Condylar histopathological staining of *Dspp*^+/–^ mice demonstrated that the cartilages over subchondral bone became remarkably thin with age. A cross-sectional study with human patients explored that low condylar bone quality was significantly correlated with TMJ OA development [7].

Along with all the above, and the fact that DSPP plays a crucial role in bone biomineralization [16, 38], we envision that DSPP deficiency defect on subchondral bone might be a contributor to exaggerating TMJ OA pathological process. Plus, increased osteoclast activity was noted at the cartilage-bone interface. The presence of increased osteoclasts would be a direct cause of extensive subchondral bone activities. However, the mechanism underlying osteoclast increase is still unknown. Nonetheless, subchondral bone resorption is a superposition effect of DSPP deficiency and osteoclast upregulation.

Both IL-6 and MMP-2 are important inflammatory agents associated with OA. Our data confirmed the increased expression of both molecules in the MCC of *Dspp*^+/–^ mice in comparison with WT mice. As mentioned above, TLRs are activated in the presence of DAMPs which have been implicated in mediating cartilage degradation [39]. We selected FN-1 as representative DAMP because FN-1 expression increased in bone in *Dspp* null mice compared with WT mice [16]. Data showed that FN-1 and its receptor TLR4 markedly increased in MCC. Nevertheless, the underlying mechanism needs further study.

*Dspp*^*−/−*^ mice exhibited defective mineralization in long bone, alveolar bone as well as skull bone [16, 17, 38]. Besides the subchondral bone, *Dspp*^+/–^ mice also bear the low quality of bone in the whole body, including alveolar bone [20] and long bone (data not shown). *Dspp* mutation, in the heterozygous background, in patients with DGI have compromised alveolar bone cells [40]. Therefore, we propose that patients with DGI II, DGI III, or DD II who carry *Dspp* mutation may be inclined to TMJ OA. Of course, clinical screening is necessary to check out the TMJ fitness in these populations.

​The drawback of this manuscript is the limitation of the sample size. In summary, this study established an animal model for inducing TMJ-OA using a single factor. However, mechanisms underlying the pathogenesis of TMJ-OA by utilizing heterozygous *Dspp* knockout mice still need in-depth exploration. Besides, this model will highlight the necessity to clinically screen patients with the *Dspp* mutations.

## Conclusion

*Dspp*^+/–^ mice exhibit TMJ OA in a time-dependent manner, with lesions in the mandibular condyle attributed to hypomineralization of subchondral bone and mandibular condylar cartilage breakdown.

### Supplementary Information

Supplementary Material 1.

## Data Availability

All RNA-seq datasets are available through the GEO database under accession code “GSE261528”. The datasets analysed during the current study are available in the ClinVar repository (https://www.ncbi.nlm.nih.gov/clinvar/submitters/509028/). The datasets used for the current study are available from the corresponding author on reasonable request.

## References

[1] Embree M, Ono M, Kilts T, Walker D, Langguth J, Mao J, Bi Y, Barth JL, Young M (2011). Role of subchondral bone during early-stage experimental TMJ osteoarthritis. J Dent Res.

[2] Glasson SS, Askew R, Sheppard B, Carito B, Blanchet T, Ma HL, Flannery CR, Peluso D, Kanki K, Yang Z (2005). Deletion of active ADAMTS5 prevents cartilage degradation in a murine model of osteoarthritis. Nature.

[3] Krisjane Z, Urtane I, Krumina G, Neimane L, Ragovska I (2012). The prevalence of TMJ osteoarthritis in asymptomatic patients with dentofacial deformities: a cone-beam CT study. Int J Oral Maxillofac Surg.

[4] Tanaka E, Detamore MS, Mercuri LG (2008). Degenerative disorders of the temporomandibular joint: etiology, diagnosis, and treatment. J Dent Res.

[5] Shirakura M, Kram V, Robinson J, Sikka S, Kilts TM, Wadhwa S, Young MF (2017). Extracellular matrix mediates BMP-2 in a model of temporomandibular joint osteoarthritis. Cells Tissues Organs.

[6] Zhen G, Wen C, Jia X, Li Y, Crane JL, Mears SC, Askin FB, Frassica FJ, Chang W, Yao J (2013). Inhibition of TGF-β signaling in mesenchymal stem cells of subchondral bone attenuates osteoarthritis. Nat Med.

[7] Shi J, Lee S, Pan HC, Mohammad A, Lin A, Guo W, Chen E, Ahn A, Li J, Ting K (2017). Association of condylar bone quality with TMJ osteoarthritis. J Dent Res.

[8] Embree MC, Kilts TM, Ono M, Inkson CA, Syed-Picard F, Karsdal MA, Oldberg A, Bi Y, Young MF (2010). Biglycan and fibromodulin have essential roles in regulating chondrogenesis and extracellular matrix turnover in temporomandibular joint osteoarthritis. Am J Pathol.

[9] Wadhwa S, Embree M, Ameye L, Young MF (2005). Mice deficient in biglycan and fibromodulin as a model for temporomandibular joint osteoarthritis. Cells Tissues Organs.

[10] Wadhwa S, Embree MC, Kilts T, Young MF, Ameye LG (2005). Accelerated osteoarthritis in the temporomandibular joint of biglycan/fibromodulin double-deficient mice. Osteoarthritis Cartilage.

[11] Hu K, Xu L, Cao L, Flahiff CM, Brussiau J, Ho K, Setton LA, Youn I, Guilak F, Olsen BR (2006). Pathogenesis of osteoarthritis-like changes in the joints of mice deficient in type IX collagen. Arthritis Rheum.

[12] Weng Y, Liu Y, Du H, Li L, Jing B, Zhang Q, Wang X, Wang Z, Sun Y (2017). Glycosylation of DMP1 is essential for chondrogenesis of condylar cartilage. J Dent Res.

[13] Sun Y, Gandhi V, Prasad M, Yu W, Wang X, Zhu Q, Feng JQ, Hinton RJ, Qin C (2010). Distribution of small integrin-binding ligand, N-linked glycoproteins (SIBLING) in the condylar cartilage of rat mandible. Int J Oral Maxillofac Surg.

[14] Sreenath T, Thyagarajan T, Hall B, Longenecker G, D'Souza R, Hong S, Wright JT, MacDougall M, Sauk J, Kulkarni AB (2003). Dentin sialophosphoprotein knockout mouse teeth display widened predentin zone and develop defective dentin mineralization similar to human dentinogenesis imperfecta type III. J Biol Chem.

[15] Qin C, Brunn JC, Cadena E, Ridall A, Butler WT (2003). Dentin sialoprotein in bone and dentin sialophosphoprotein gene expressed by osteoblasts. Connect Tissue Res.

[16] Chen Y, Zhang Y, Ramachandran A, George A (2016). DSPP is essential for normal development of the dental-craniofacial complex. J Dent Res.

[17] Gibson MP, Zhu Q, Liu Q, D'Souza RN, Feng JQ, Qin C (2013). Loss of dentin sialophosphoprotein leads to periodontal diseases in mice. J Periodontal Res.

[18] Liu Q, Gibson MP, Sun H, Qin C (2013). Dentin sialophosphoprotein (DSPP) plays an essential role in the postnatal development and maintenance of mouse mandibular condylar cartilage. J Histochem Cytochem.

[19] MacDougall M, Simmons D, Luan X, Nydegger J, Feng J, Gu TT (1997). Dentin phosphoprotein and dentin sialoprotein are cleavage products expressed from a single transcript coded by a gene on human chromosome 4. Dentin phosphoprotein DNA sequence determination. J Biol Chem.

[20] Shi C, Ma N, Zhang W, Ye J, Shi H, Xiang D, Wu C, Song L, Zhang N, Liu Q (2020). Haploinsufficiency of Dspp gene causes dentin dysplasia type II in mice. Front Physiol.

[21] Verdelis K, Szabo-Rogers HL, Xu Y, Chong R, Kang R, Cusack BJ, Jani P, Boskey AL, Qin C, Beniash E (2016). Accelerated enamel mineralization in Dspp mutant mice. Matrix Biol.

[22] Matías EM, Mecham DK, Black CS, Graf JW, Steel SD, Wilhelm SK, Andersen KM, Mitchell JA, Macdonald JR, Hollis WR (2016). Malocclusion model of temporomandibular joint osteoarthritis in mice with and without receptor for advanced glycation end products. Arch Oral Biol.

[23] Coiner-Collier S, Vogel ER, Scott RS (2018). Trabecular anisotropy in the primate mandibular condyle is associated with dietary toughness. Anat Rec (Hoboken).

[24] Roh JS, Sohn DH (2018). Damage-associated molecular patterns in inflammatory diseases. Immune Netw.

[25] Carballo CB, Nakagawa Y, Sekiya I, Rodeo SA (2017). Basic science of articular cartilage. Clin Sports Med.

[26] Chen PJ, Dutra EH, Mehta S, O'Brien MH, Yadav S (2020). Age-related changes in the cartilage of the temporomandibular joint. Geroscience.

[27] Zhao Y, An Y, Zhou L, Wu F, Wu G, Wang J, Chen L (2022). Animal models of temporomandibular joint osteoarthritis: classification and selection. Front Physiol.

[28] Cui C, Zheng L, Fan Y, Zhang J, Xu R, Xie J, Zhou X (2020). Parathyroid hormone ameliorates temporomandibular joint osteoarthritic-like changes related to age. Cell Prolif.

[29] Rahmati M, Nalesso G, Mobasheri A, Mozafari M (2017). Aging and osteoarthritis: central role of the extracellular matrix. Ageing Res Rev.

[30] Ameye L, Aria D, Jepsen K, Oldberg A, Xu T, Young MF (2002). Abnormal collagen fibrils in tendons of biglycan/fibromodulin-deficient mice lead to gait impairment, ectopic ossification, and osteoarthritis. FASEB J.

[31] Fässler R, Schnegelsberg PN, Dausman J, Shinya T, Muragaki Y, McCarthy MT, Olsen BR, Jaenisch R (1994). Mice lacking alpha 1 (IX) collagen develop noninflammatory degenerative joint disease. Proc Natl Acad Sci U S A.

[32] Huang K, Wu LD (2008). Aggrecanase and aggrecan degradation in osteoarthritis: a review. J Int Med Res.

[33] Hyttinen MM, Töyräs J, Lapveteläinen T, Lindblom J, Prockop DJ, Li SW, Arita M, Jurvelin JS, Helminen HJ (2001). Inactivation of one allele of the type II collagen gene alters the collagen network in murine articular cartilage and makes cartilage softer. Ann Rheum Dis.

[34] Xu L, Flahiff CM, Waldman BA, Wu D, Olsen BR, Setton LA, Li Y (2003). Osteoarthritis-like changes and decreased mechanical function of articular cartilage in the joints of mice with the chondrodysplasia gene (cho). Arthritis Rheum.

[35] Sun Y, Ma S, Zhou J, Yamoah AK, Feng JQ, Hinton RJ, Qin C (2010). Distribution of small integrin-binding ligand, N-linked glycoproteins (SIBLING) in the articular cartilage of the rat femoral head. J Histochem Cytochem.

[36] McKnight DA, Simmer JP, Hart PS, Hart TC, Fisher LW (2008). Overlapping DSPP mutations cause dentin dysplasia and dentinogenesis imperfecta. J Dent Res.

[37] Nieminen P, Papagiannoulis-Lascarides L, Waltimo-Siren J, Ollila P, Karjalainen S, Arte S, Veerkamp J, Tallon Walton V, Chimenos Küstner E, Siltanen T (2011). Frameshift mutations in dentin phosphoprotein and dependence of dentin disease phenotype on mutation location. J Bone Miner Res.

[38] Verdelis K, Ling Y, Sreenath T, Haruyama N, MacDougall M, van der Meulen MC, Lukashova L, Spevak L, Kulkarni AB, Boskey AL (2008). DSPP effects on in vivo bone mineralization. Bone.

[39] Cafferata EA, Monasterio G, Castillo F, Carvajal P, Flores G, Díaz W, Fuentes AD, Vernal R (2021). Overexpression of MMPs, cytokines, and RANKL/OPG in temporomandibular joint osteoarthritis and their association with joint pain, mouth opening, and bone degeneration: a preliminary report. Oral Dis.

[40] Porntaveetus T, Nowwarote N, Osathanon T, Theerapanon T, Pavasant P, Boonprakong L, Sanon K, Srisawasdi S, Suphapeetiporn K, Shotelersuk V (2019). Compromised alveolar bone cells in a patient with dentinogenesis imperfecta caused by DSPP mutation. Clin Oral Investig.

