# Supplementary material for: Long-term haplodeficency of DSPP causes temporomandibular joint osteoarthritis in mice
Source: BMC Oral Health. 2024 May 14;24:569. doi: 10.1186/s12903-024-04320-8 (PMC11094853; doi:10.1186/s12903-024-04320-8)
Supplement: Supplementary file 1 — Supplementary Material 1. [file 12903_2024_4320_MOESM1_ESM.docx]

**Additional files 1:**

**1.**Following our analysis of the differentially expressed genes obtained from RNA-seq, we conducted GO and KEGG analyses. The results of our study revealed the enrichment of certain pathways related to osteogenesis, including a downregulation of genes associated with osteogenesis and an upregulation of genes linked to osteoclast differentiation in *Dspp^+/-^* mice. These results suggest the involvement of underlying mechanisms that contribute to the regulation of osteogenesis and osteoclast differentiation.


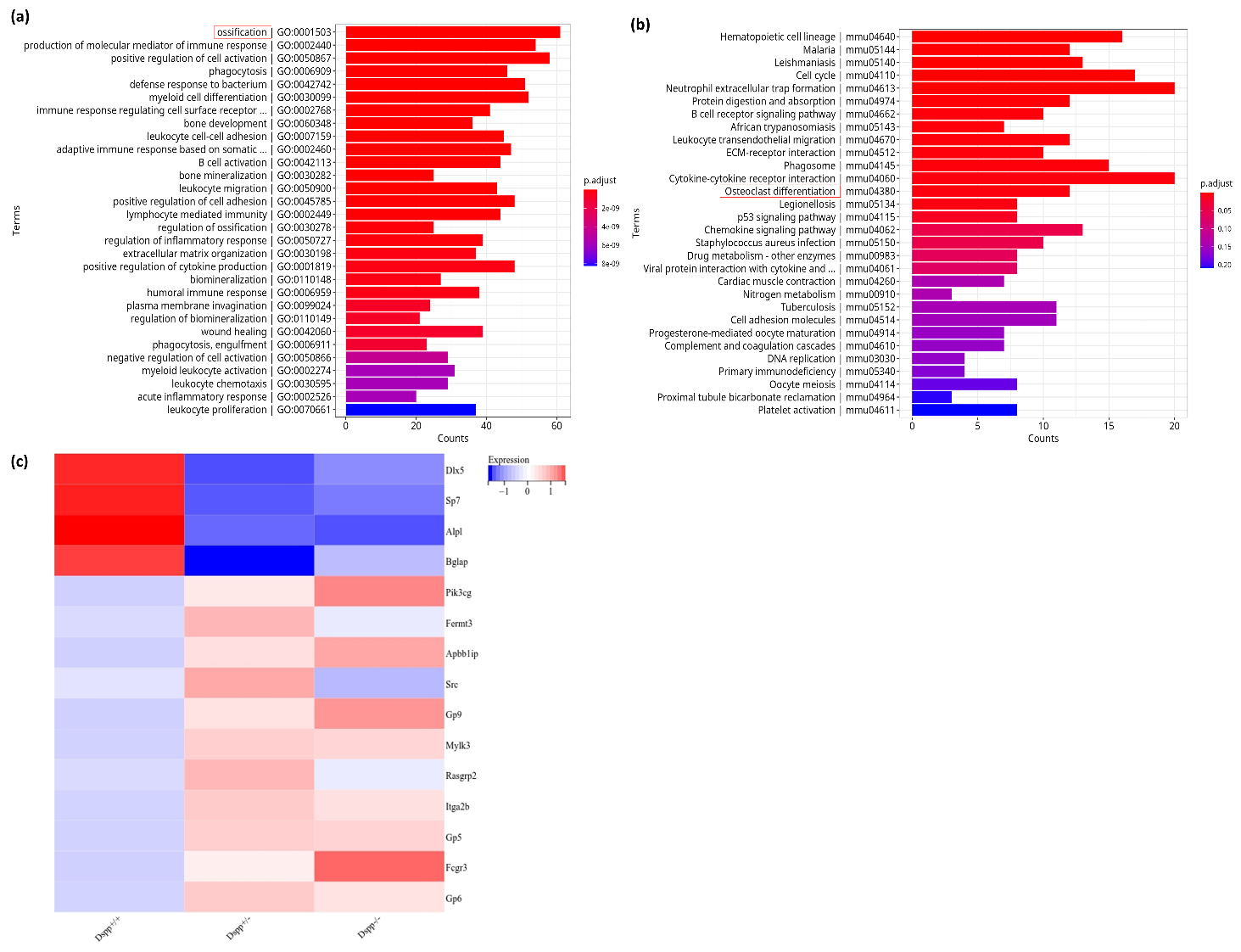
**Fig. S1** Haplodeficency of DSPP affects the biological processes of ossification and osteoclast differentiation. (a) The top 30 Gene ontology (GO) biological process enriched in the comparison between condyles in WT mice and *Dspp^+/-^* mice. (b) Genes annotated for the KEGG pathway enriched in the WT and *Dspp^+/-^* mouse condyles. (c) Heatmap of representative osteogenesis and osteoclast differentiation genes in WT, *Dspp^+/-^* and *Dspp^-/-^* mice.

2. It is evident from the immunohistochemistry of mice condyle that there is a reduction in DSPP in *Dspp^+/-^* mice.


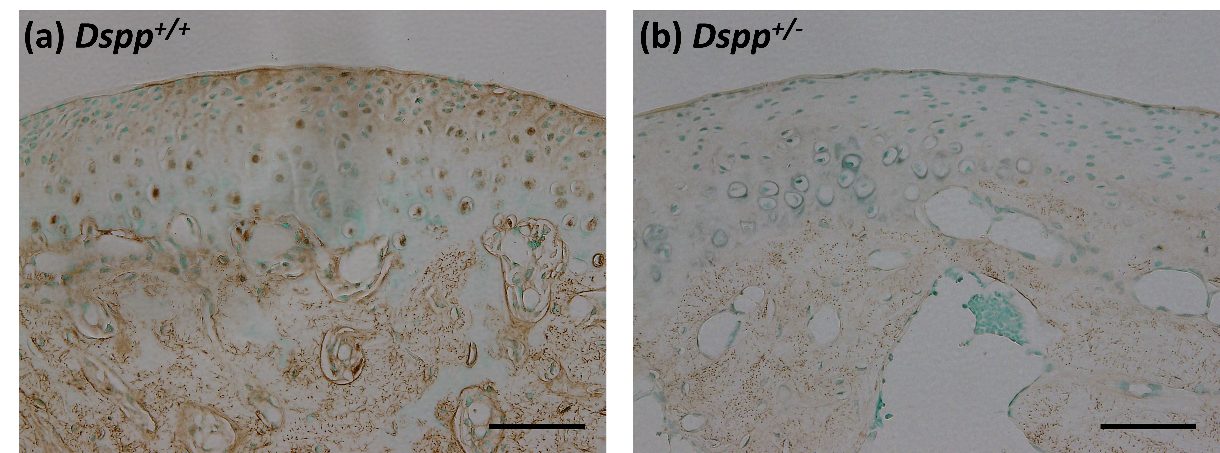


**Fig. S2** Expression of DSPP in the mandibular condylar cartilage. (a, b) Images of immunohistochemical staining in the mandibular condylar cartilage of both WT mice and *Dspp^+/-^* mice at the age of 6 months. The data show that there is a positive DSPP reaction in both the cartilage and subchondral bone in WT mice. However, in *Dspp^+/-^* mice, the positive signal is marked weaker. The scale bar in the image is 50 μm.
